# Dynamic muscle loss and metabolic dysregulation in sepsis-associated muscle wasting: an integrated ultrasound and metabolomics study

**DOI:** 10.3389/fmed.2026.1868564

**Published:** 2026-07-23

**Authors:** Jinlan Ma, Jinyuan Li, Xuepeng Bai, Hanyu Mai, Tao Feng, Jianjun Yang, Huan Ding

**Affiliations:** 1 Department of Critical Medicine, General Hospital of Ningxia Medical University, Yinchuan, China; 2 Clinical Medical College of Ningxia Medical University, Yinchuan, China; 3 Cardiovascular Surgery, General Hospital of Ningxia Medical University, Yinchuan, China; 4 Ningxia Integrated Traditional Chinese and Western Medicine Hospital, Yinchuan, China; 5 School of Public Health, Ningxia Medical University, Yinchuan, China

**Keywords:** bedside ultrasonography, metabolomics, rectus femoris, sepsis-associated muscle wasting, texture features

## Abstract

**Objective:**

This study aimed to investigate early changes in the rectus femoris muscle during sepsis-associated muscle wasting (SAMW) and their association with metabolic dysregulation through a dynamic analysis of ultrasound texture features and metabolomics.

**Methods:**

In this prospective observational study, adults with sepsis underwent ultrasound of the right rectus femoris on intensive care unit (ICU) days 1, 4, and 7 to measure rectus femoris cross-sectional area (RF-CSA), muscle layer thickness (RF-MLT), and gray-level co-occurrence matrix texture features. Linear mixed-effects models were used to assess group, time, and group-by-time effects, and repeated-measures correlations were used to test longitudinal associations with metabolic/inflammatory biomarkers. Untargeted metabolomics was performed in the SAMW subgroup.

**Results:**

Of the 751 patients screened, 60 were included, and 16 (26.7%) developed SAMW. A significant group-by-time interaction was observed only for RF-CSA (*p* = 0.031), which declined in SAMW (slope −0.121, SE 0.039; *p* = 0.0024). Patients with sepsis showed reduced systemic inflammation during the first week in the ICU; however, SAMW featured persistent muscle wasting, possibly driven by ongoing glucose/lipid dysregulation and hypercatabolism. RF-MLT was negatively correlated with low-density lipoprotein (*r* = −0.42, *p* < 0.001), and RF-CSA with total cholesterol (*r* = −0.25, *p* < 0.05). Texture standard deviation correlated positively with glucose (*r* = 0.29, *p* < 0.01) and negatively with interleukin-6 (*r* = −0.23, *p* < 0.05). Metabolomics identified 29 metabolites that progressively decreased from controls to SAMW on days 1 and 7, with enrichment of fatty acid pathways; docosahexaenoic acid and arachidonic acid emerged as key differential metabolites.

**Conclusion:**

Although systemic inflammation gradually decreased during the first week of ICU stay in patients with sepsis, those who developed SAMW showed persistent loss of muscle mass associated with metabolic dysregulation.

**Clinical trial registration:**

The study was registered at ClinicalTrials.gov (registration no.: ChiCTR2400092420).

## 1 Introduction

Approximately 30% of patients admitted to the intensive care unit (ICU) have sepsis, which is associated with high mortality (1, 2). Nearly one-third of the patients with sepsis die within 30 days, and this risk is closely linked to disease-related complications (3). Sepsis-associated muscle wasting (SAMW), also known as ICU-acquired weakness (ICUAW), is a major complication of sepsis, characterized by the loss of skeletal muscle mass, myofiber atrophy, and reduced muscle strength. The rectus femoris is one of the most frequently assessed and best-characterized muscles in ICUAW (4–6). In this study, ultrasound texture features combined with metabolomics to longitudinally characterize changes in the rectus femoris were used during week 1 of ICU stay in patients with sepsis, and investigated their association with metabolic dysregulation.

Ultrasound is a safe and non-invasive tool for quantifying muscle cross-sectional area and thickness, making it particularly suitable for bedside assessment (7). Texture features derived from spatial variations in ultrasound pixel intensities, commonly quantified using the gray-level co-occurrence matrix (GLCM) method, can capture intrinsic tissue heterogeneity that is not readily visible to the naked eye (8). Previous studies have focused primarily on diabetes and chronic kidney disease (9, 10), whereas evidence in the rapidly evolving high-risk sepsis population remains scarce.

A hallmark of sepsis is the dysregulation of cellular bioenergetics and metabolism (11). As a key metabolic organ, skeletal muscle provides an accessible window for bedside monitoring. Metabolomics can capture rapid dynamic changes at the small-molecule level and has been increasingly applied in the diagnosis and treatment of sepsis (12, 13). However, most of the existing evidence is cross-sectional or limited to one-dimensional measures. Therefore, this study integrated longitudinal bedside ultrasound texture features, serial monitoring of clinical metabolic and inflammatory biomarkers, and untargeted metabolomics to characterize the dynamic changes in SAMW and investigate their association with metabolic dysregulation.

## 2 Methods

### 2.1 Study design and participants

In this prospective cohort study, 1,252 patients admitted to the ICU of the General Hospital of Ningxia Medical University in China (a tertiary teaching hospital) between October 2022 and August 2023 were screened. Among these, 751 met the Sepsis-3 criteria. After exclusion according to the prespecified criteria, 60 patients with sepsis were included in the final analysis. The inclusion criteria were as follows: (1) diagnosised of sepsis within 24 h, according to the “Sepsis-3.0” definition (1). The exclusion criteria were as follows: (1) pregnancy, (2) age < 18 years, (3) traumatic brain injury or cervical spinal cord injury, (4) bilateral lower limb fractures, (5) history of neuromuscular diseases, and (6) unwillingness to participate. Nutritional therapy was administered according to the 2019 ESPEN ICU Clinical Nutrition Guidelines (14).

### 2.2 Ultrasound imaging acquisition

On ICU days 1, 4, and 7, a trained ICU physician with bedside ultrasound certification and 5 years of experience performed standardized scanning using a portable color Doppler ultrasound system (M9super, Mindray, Shenzhen, China). The right rectus femoris muscle layer thickness (RF-MLT) and cross-sectional area (RF-CSA) were measured with patients in the supine position. Ultrasound scanning was conducted in the upper third of the right patella using a linear array probe in B-mode. The gain setting was adjusted between 40 and 70% to optimize the image brightness while minimizing probe pressure on the leg of the patient (15). The ultrasound beam was aligned perpendicularly to the femoral shaft to visualize the rectus femoris muscle. Each measurement was repeated thrice and the average value was recorded. To ensure consistency, measurement sites were marked for subsequent scans. The ultrasound operator was blinded to the clinical data of the patient and did not participate in the treatment. Reductions of 20% in RF-MLT or 10% in RF-CSA were used to assign patients to the SAMW group, whereas patients not meeting these criteria were assigned to the Non-sepsis-associated muscle wasting (NSAMW) group (16, 17).

### 2.3 Texture feature extraction

Texture analysis was performed using the Texture Analysis System (AnTex; Mindray), that supports annotation, measurement, and texture feature extraction. A second ICU physician with 15 years of experience, blinded to the patient information, conducted GLCM-based texture analysis (8 gray levels; step size 1; four directions 0°/45°/90°/135°averaged) (18). Five classic features were extracted: standard deviation (SDev), entropy (ENT), variance (VAR), homogeneity (HOM), and contrast (CON).

### 2.4 Untargeted metabolomics analysis

Untargeted metabolomics was performed in the SAMW subgroup (n = 16). Healthy volunteers matched for baseline characteristics (age, sex, body mass index) were recruited as controls (n = 12). Peripheral plasma was collected from patients with SAMW on ICU days 1 and 7 and from controls, yielding three groups: SAMW Day 1, SAMW Day 7, and control.

Metabolomics profiling was performed using UHPLC (Vanquish, Thermo Fisher, Germany) coupled with high-resolution Q Exactive™ HF-X mass spectrometry (Thermo Fisher, Germany) (LC–MS/MS). Data were acquired in both positive and negative ion modes to improve metabolite coverage. Data processing used Discoverer 3.3 (Thermo Fisher) with filtering by retention time and m/z. Peak areas were corrected using the first QC sample, and peak extraction applied a mass deviation of 5 ppm and a signal intensity deviation of 30%, along with minimum signal intensity and adduct information. Background ions were removed using blank samples. The quantification results were normalized to obtain the relative peak areas, and compounds with a QC CV of > 30% were excluded. The metabolites were annotated using the KEGG, HMDB, and LIPIDMaps databases. Multivariate analysis (principal component analysis [PCA] and partial least squares-discriminant analysis [PLS-DA]) was performed using MetaX after data transformation. The variable importance in projection (VIP) was derived using PLS-DA. Univariate analysis was performed using t-tests to compute *p*-values and fold changes (FC). Differentially expressed metabolites (DEMs) were defined as VIP > 1.0, *p* < 0.05, and FC ≥ 1.2 or FC ≤ 0.833. KEGG pathway enrichment used *p* < 0.05 as the significance threshold.

### 2.5 Clinical data collection

The following clinical data were collected - Epidemiology and medical history: Age, sex, BMI, basal metabolic rate (BMR), acute physiology and chronic health evaluation (APACHE II score), sequential organ failure assessment (SOFA score), and Nutrition Risk in Critically Ill (NUTRIC) score within 24 h of ICU admission. Outcomes: ICU length of stay, mechanical ventilation duration, and 28-day mortality. Laboratory indicators (ICU days 1, 4, and 7) included glucose metabolism, C-peptide (CP), insulin, and random glucose (GLU). Lipid metabolism: total cholesterol (TC), triglyceride, high-density lipoprotein (HDL), and low-density lipoprotein (LDL). Protein metabolism: Retinol-binding protein (RBP), transferrin (TRF), ceruloplasmin (CER), and urea-to-creatinine ratios (UCR). Inflammation: high-sensitivity C-reactive protein (hs-CRP), interleukin-6 (IL-6).

### 2.6 Statistical methods

All analyses were conducted using R software (v4.2.2). Continuous variables were tested for normality and homogeneity of variance and summarized as mean ± SD or median (Q1, Q3), as appropriate. Longitudinal trajectories were modeled using Linear mixed-effects models(LMMs) with time as a continuous fixed effect, with group, time, and group-by-time interaction included as fixed effects and patient identity included as a random intercept. APACHE II score, age, ICU length of stay, and duration of mechanical ventilation were included as prespecified covariates in the model. The model equation was: Yij = β0 + β1(Time) + β2(Group) + β3(Group×Time) + β4(Age) + β5(ICU residence time) + β6(APACHE II) + β7(Mechanical ventilation time) + ui + εij, where ui is the patient-specific random intercept, reflecting between-patient variability in baseline values; andεij is the residual error. Fixed effects and interactions were assessed using likelihood-ratio tests, and clinically meaningful covariates were pre-specified to reduce confounding. Repeated-measures correlation was used to estimate within-subject associations between ultrasound parameters and metabolic/inflammatory markers. Two-sided *p* < 0.05 was considered significant (*p < 0.05, **p < 0.01, and ***p < 0.001). Missing data were mainly due to ICU discharge before follow-up assessments in six patients and death before day 7 in two patients. The primary missing data mechanism was considered missing at random (MAR) because missingness was closely related to the patients’ observed clinical status. Therefore, longitudinal analyses were performed using LMMs fitted by maximum likelihood estimation. This prospective cohort study based its sample size on Yao and colleagues prospective ICU cohort (19), the RF-CSA atrophy rate between day 1 and day 3 was 7.9 ± 2.8% vs. 4.3 ± 2.1%, assuming a mean difference of 3.6%, a conservative SD of 2.8%, a two-sided *α* of 0.05, and 80% power, with at least 10 patients per group required. Allowing for 10% missing or incomplete data, the minimum sample size was set at 12 per group, or 24 in total. Ultimately 28 patients (16 in the SAMW group and 12 in the control group) were included in the metabolomic analysis, exceeding the minimum requirement.

## 3 Results

### 3.1 Baseline characteristics

**Figure 1:**
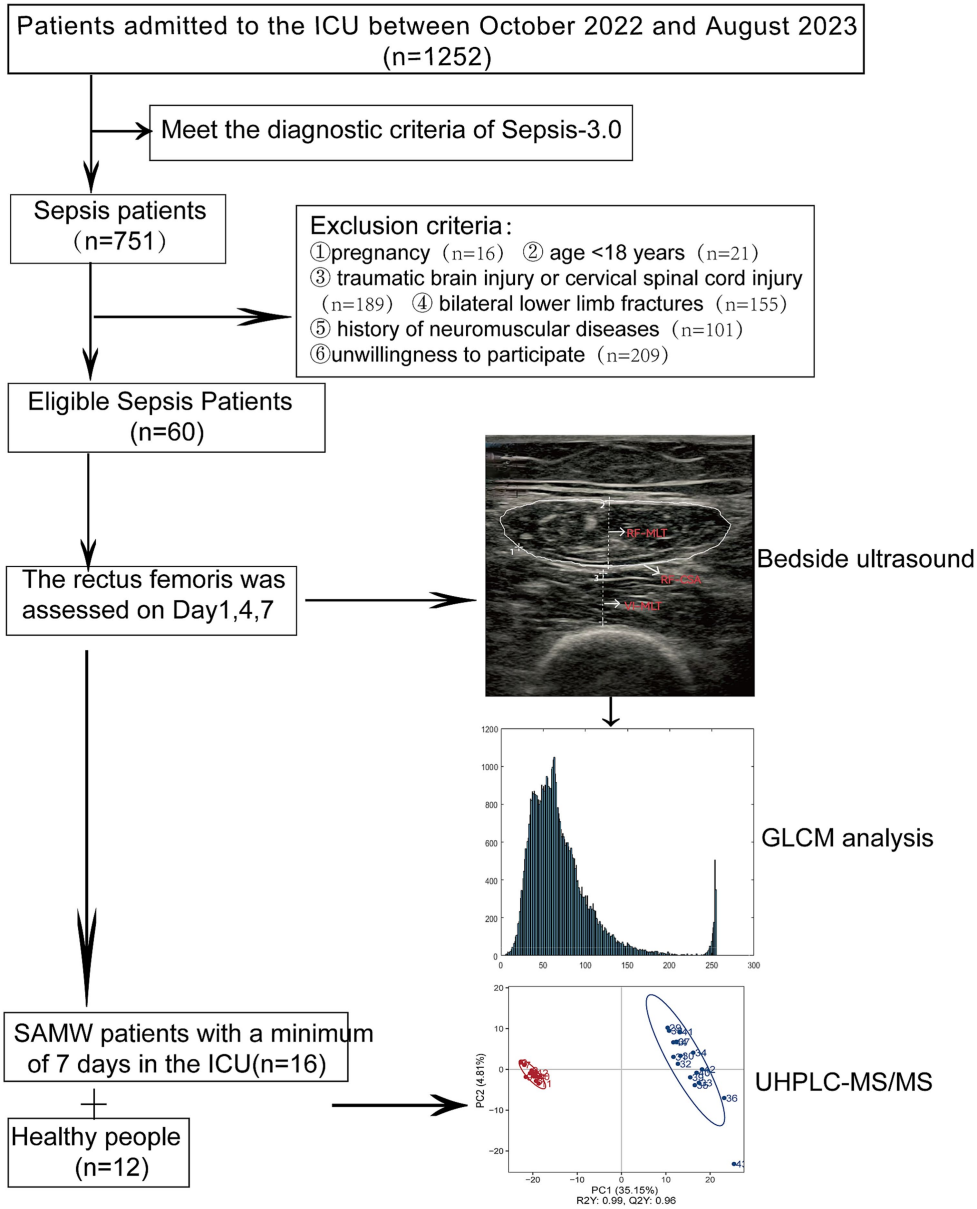
Flow chart of patient screening.

Of 751 patients screened for sepsis, 60 met the inclusion criteria. SAMW developed in 16 patients (26.7%), whereas 44 (73.3%) were classified as having non-SAMW. There were no significant between-group differences in the demographics (sex, age, BMI, and BMR) or severity scores (APACHE II and SOFA) (all *p* > 0.05). ICU length of stay was significantly longer in the SAMW group than in the non-SAMW group [11.0 days (IQR 9.0–15.0) vs. 5.0 days (IQR 4.0–8.0), *p* < 0.001]. The duration of mechanical ventilation was also longer in the SAMW group [240.0 h (IQR 132.0–328.5) vs. 45.0 h (IQR 14.5–136.5), *p* = 0.005] (Table 1). The patient screening flowchart is shown in Figure 1.

**Table 1:** Characteristics of patients.

Characteristics	Total (*n* = 60)	SAMW (*n* = 16)	NSAMW (*n* = 44)	*x*^2^/Z/t	*p*
Male [case (%)]	34 (56.7)	9 (56.3)	25 (56.8)	0.002	0.969
age (year)^a^	63.0 ± 14.1	66.0 ± 7.3	62.8 ± 15.4	9.125	0.303
BMI (kg/㎡)^a^	23.23 ± 3.22	23.92 ± 3.15	23.11 ± 3.40	0.446	0.348
BMR (kcal)^a^	1294.03 ± 191.72	1298.75 ± 178.38	1292.27 ± 198.64	0.303	0.912
APACHEII(score)^b^	15.0 (12.3,20.8)	16.0 (13.0,22.0)	15.5 (13.0,20.5)	0.704	0.481
SOFA (score)^b^	7.0 (5.0,9.0)	7.0 (5.0,8.5)	7 (5.0,9.0)	−0.379	0.705
NUTRIC(score)^b^	4.0 (3.0,5.8)	5.0 (3.5,5.3)	4.0 (3.0,6.0)	0.485	0.628
ICU residence time(d) ^b^	7.0 (4.0,12.5)	11.0 (9.0,15.0)	5.0 (4.0,8.0)	3.531	<0.001
Mechanical ventilation time (h)^b^	66.0 (11.3,211.0)	240.0 (132.0,328.5)	45.0 (14.5,136.5)	2.803	0.005
Septic shock [*n* (%)]	32 (53.3)	8 (50.0)	24 (54.5)	0.097	0.755
28-day mortality rate [*n* (%)]	11 (18.3)	4 (25.0)	7 (15.9)	0.648	0.421

BMI, Body mass index; BMR, basal metabolic rate; APACHE II, Acute Physiology and Chronic Health Status II; SOFA, Sequential Organ failure; NUTRIC, Nutrition Risk in the Critically Ill; ^a^is mean ± SD and b is median (Q1, Q3).

### 3.2 Longitudinal changes in muscle ultrasound parameters and associations with metabolic/inflammatory markers

**Figure 2:**
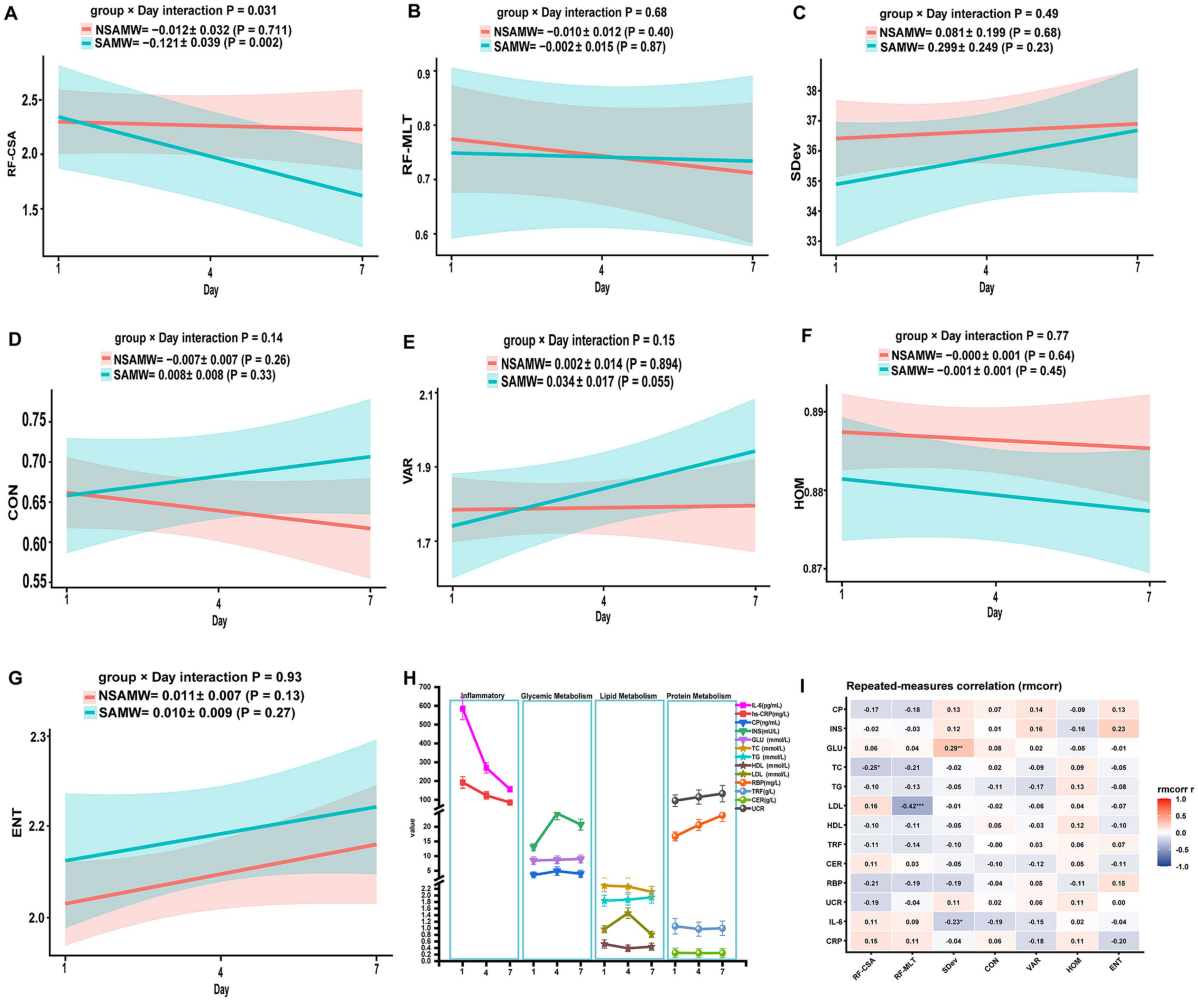
Ultrasound texture features. **(A–G)** Muscle texture features on days 1, 4, and 7 in the NSAMW and SAMW groups. Lines show estimated marginal means; shaded areas show 95% CIs. Group × day interaction *p* values are shown in each panel. **(H)** Changes in inflammatory, glycaemic, lipid, and protein metabolism biomarkers. Data are means ± SD. **(I)** Repeated-measures correlation heatmap. Color indicates correlation direction and strength; asterisks indicate significance (*p < 0.05, **p < 0.01, ***p < 0.001).

Using LMMs, the trajectories of muscle ultrasound texture features across the ICU on days 1, 4, and 7 were assessed. The RF-CSA was the only variable that exhibited a significant group-by-time interaction (*p* = 0.031). In the SAMW group, RF-CSA declined significantly over time (slope −0.121, SE 0.039; *p* = 0.0024), whereas no significant change was observed in the non-SAMW group (slope −0.012, SE 0.032; *p* = 0.71; Figure 2A), VAR exhibited a non-significant upward trend (slope = 0.034, SE = 0.017; *p* = 0.055), but the group-by-time interaction was not significant (*p* = 0.15; Figure 2E). Other texture features did not show significant group-specific temporal changes or interactions (all interactions, *p* > 0.05) (Figures 2B−D,F,G).

Thirteen key biomarkers of inflammation and glucose, lipid, and protein metabolism were also monitored at the same time points (Figure 2H). Overall, inflammatory markers decreased during the first week in the ICU, suggesting the attenuation of systemic inflammation with the initial treatment. In contrast, glucose metabolism markers showed decreased C-peptide and insulin levels with an upward trend in glucose levels, consistent with insulin resistance and dysregulated glycemic control in sepsis. Protein metabolism showed increased UCR (catabolism) and decreased TRF and CER (anabolism) levels, indicating sustained hypercatabolic and hypoanabolic states. Lipid indices showed reduced TC and HDL, with increased LDL levels around ICU day 4, suggesting characteristic lipid remodeling in early sepsis. Thus, although the systemic inflammation improved, a metabolic crisis characterized by dysglycemia, lipid alterations, and enhanced proteolysis persisted.

A repeated-measures correlation demonstrated a moderate negative association between RF-MLT and LDL (*r* = −0.42, *p* < 0.001) and a negative association between RF-CSA and TC (*r* = −0.25, *p* < 0.05), indicating that muscle loss co-occurred with lipid abnormalities. In addition, SDev correlated positively with GLU (*r* = 0.29, *p* < 0.01) and negatively with IL-6 (*r* = −0.23, *p* < 0.05), suggesting that textural features may capture microenvironmental changes that are not synchronous with inflammatory resolution. To explore these mechanistic links, we performed an untargeted metabolomic analysis (Figure 2I).

### 3.3 Metabolomics data quality and model validation

**Figure 3:**
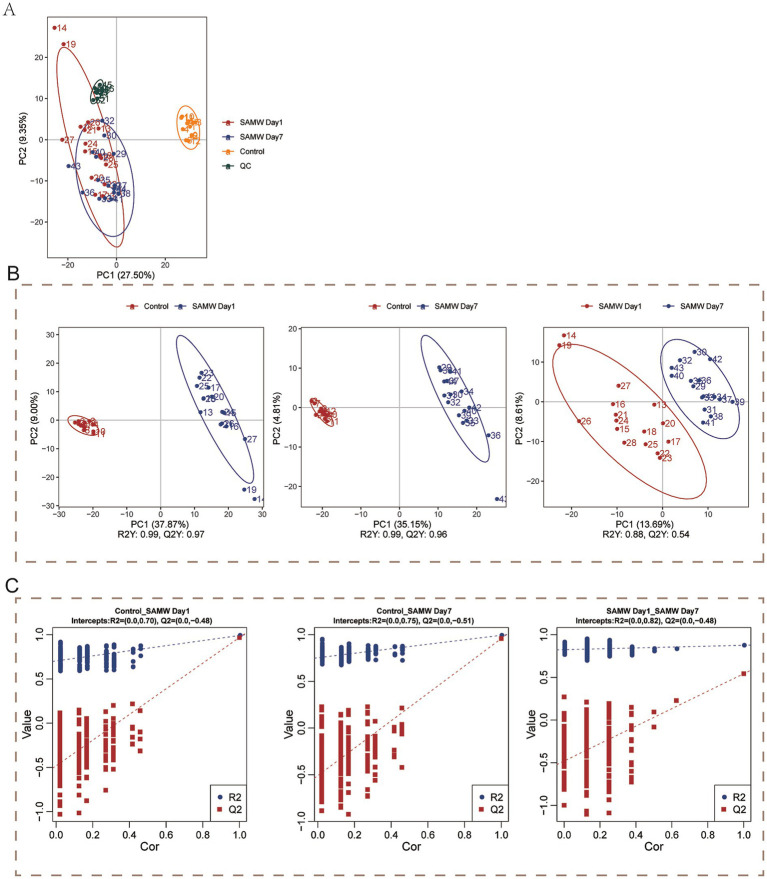
Data quality control. **(A)** PCA score plot. Dots represent samples, colors represent groups, and ellipses show 95% CIs. **(B)** PLS-DA score plot. R^2^Y and Q^2^Y indicate model explanatory and predictive performance, respectively. **(C)** PLS-DA permutation test. Each point represents one permutation, with R^2^ and Q^2^ scores shown.

After data normalization in both positive and negative ion modes, 1,038 metabolites were identified across the SAMW Day 1, Day 7, and control groups. The PCA score plot showed an unsupervised distribution of the Control, SAMW Day 1, and SAMW Day 7 samples (Figure 3A). A PLS-DA model was constructed to assess the relationship between metabolite expression levels and sample categories for predictive analysis. Pairwise PLS-DA score comparisons showed that R^2^Y values were close to 1, with R^2^Y being greater than Q^2^Y, indicating a good model fit and significant intergroup differences, ensuring the reliability of the identified metabolites (Figure 3B). The robustness of the PLS-DA model was assessed using 200 permutations. In each permutation, the sample class labels were randomly reassigned and the PLS-DA model was refitted and used for prediction to obtain the corresponding *R*^2^ and Q^2^ values. The regression lines for R^2^ and Q^2^ were evaluated. The *R*^2^ values were higher than the Q^2^ values, and the intercept of the Q^2^ regression line on the Y-axis was < 0, indicating a low risk of overfitting and supporting good model stability and predictive performance (Figure 3C).

### 3.4 Identification and visualization of differential metabolites

**Figure 4:**
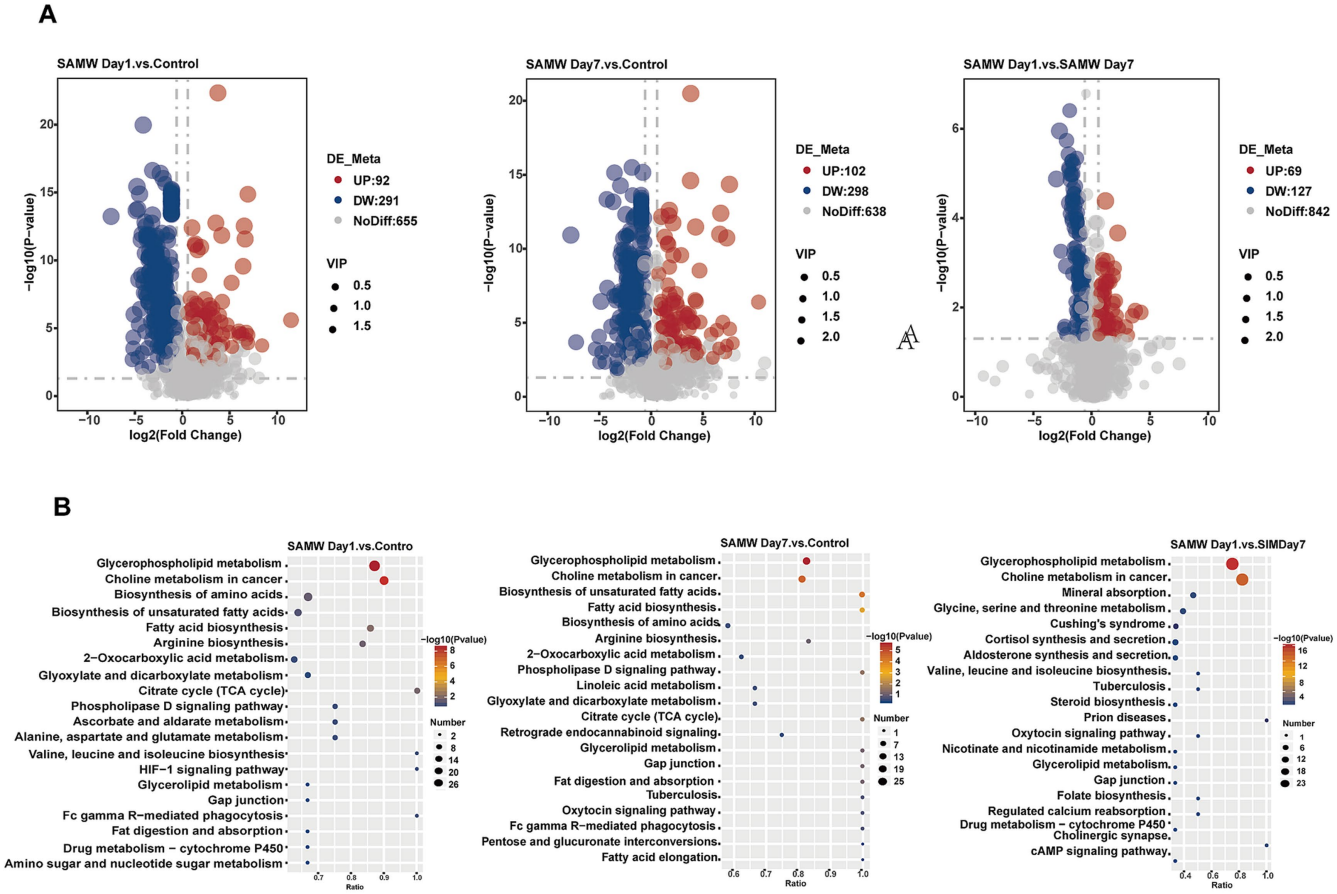
Differential metabolite analysis. **(A)** Volcano plot. Each point represents a metabolite; size indicates VIP value. Red and blue indicate upregulated and downregulated metabolites, respectively. **(B)** Top 20 KEGG enrichment bubble plot. The x-axis shows the DEM ratio per pathway. Point color indicates p value, and size indicates DEM count.

Through pairwise comparisons between SAMW days 1, 7, and control groups DEMs were identified. In the SAMW Day 1 vs. control comparison, 92 metabolites were upregulated, whereas 291 were downregulated. In the SAMW Day 7 vs. control comparison, 102 metabolites were upregulated, and 298 were downregulated. In the SAMW Day 1 vs. SAMW Day 7 comparison, 69 metabolites were upregulated and 127 were downregulated (Figure 4A). The FC of the DEMs was log-transformed (base 2) and the top 20 upregulated and downregulated metabolites were visualized in a stem plot (Figure 4B).

KEGG enrichment analysis revealed significant metabolic pathway alterations in SAMW patients. In the SAMW Day 1 vs. Control comparison, glycerophospholipid metabolism, choline metabolism in cancer, fatty acid (FA) biosynthesis, citrate cycle (TCA cycle), arginine biosynthesis, and amino acid biosynthesis were significantly affected. This suggests that upon ICU admission, the metabolic profile of patients with SAMW is primarily characterized by alterations in FA metabolism and amino acid biosynthesis. In the SAMW Day 7 vs. control comparison, pathways including glycerophospholipid metabolism, choline metabolism in cancer, biosynthesis of unsaturated fatty acids (UFA), FA biosynthesis, the TCA cycle, and the phospholipase D signaling pathway were significantly affected, indicating that FA metabolism remained the most significantly altered pathway after 7 days in the ICU. In the SAMW Day 1 vs. Day 7 comparison, glycerophospholipid metabolism and choline metabolism in cancer were the most significantly altered pathways (Figure 4C). Overall, FA metabolism was the primary metabolic pathway that was significantly altered in patients with SAMW at ICU admission and after 7 days, suggesting its critical role in the pathogenesis of SAMW.

### 3.5 Trend analysis and identification of key metabolites

One-way analysis of variance was performed to compare the data across the three groups to identify DEMs and their enriched pathways, resulting in the identification of 228 significant DEMs. A heatmap of the relative abundance of DEMs in each sample was generated through clustering analysis, and K-means clustering analysis was used to classify metabolites into distinct patterns.

**Figure 5:**
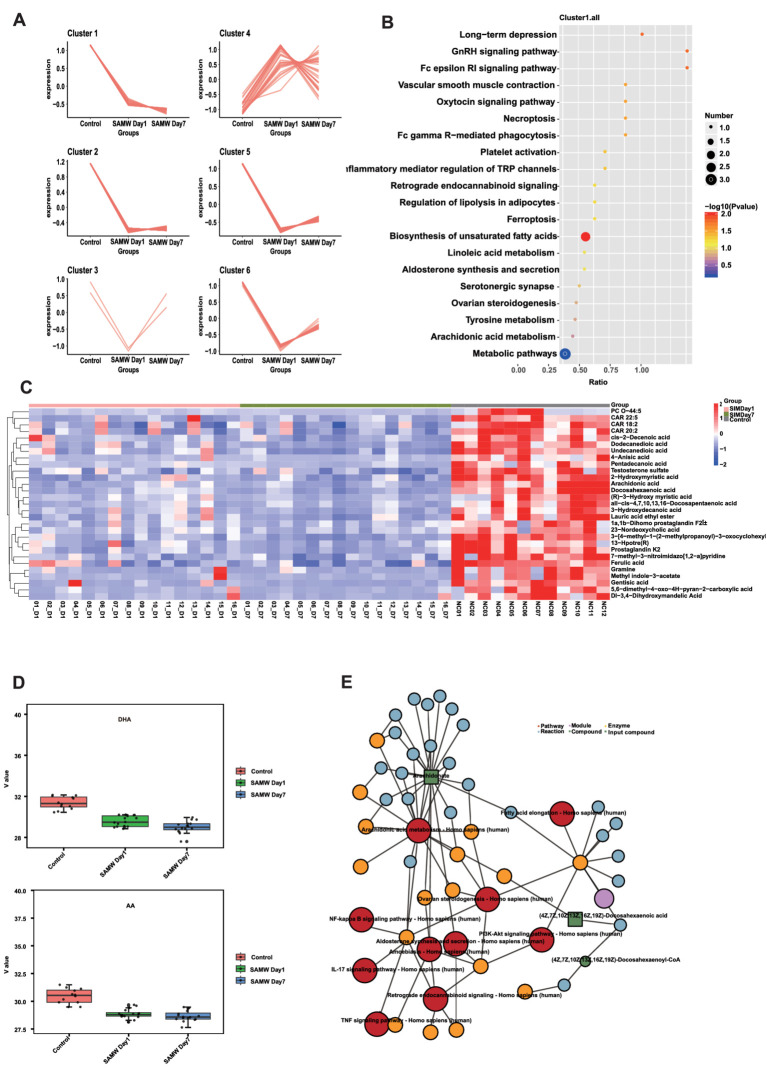
Trend analysis and Differential metabolites. **(A)** K-means clustering of z-score-normalized metabolite abundance across groups. **(B)** KEGG enrichment bubble plot. The x-axis shows the DEM ratio per pathway; point color and size indicate p value and DEM count.**(C)** Heatmap of 29 DEMs across samples.**(D)** Boxplot of metabolite levels across groups. **(E)** KEGG network showing pathways, enzymes, compounds, modules, reactions, and differential metabolites.

Because RF-CSA in the SAMW group declined significantly over time, this study focused on clusters in which the relative abundance of metabolites showed consistently increasing or decreasing trends from the Control through SAMW Day 1 to Day 7. Among the 228 DEMs, 29 exhibited a progressive downward trend corresponding to Cluster 1 (Figures 5A,B). These metabolites were significantly enriched in pathways, such as biosynthesis of UFAs, Fc epsilon RI signaling pathway, Long-term depression, GnRH signaling pathway, Necroptosis, Vascular smooth muscle contraction, Fc *γ* R-mediated phagocytosis, oxytocin signaling pathway, platelet activation, and regulation of TRP channels by inflammatory mediators. Among these, docosahexaenoic acid (DHA) and arachidonic acid (AA) were the only metabolites identified as significant DEMs (Figures 5C,D). This suggests that the onset and progression of SAMW within the first week of the ICU stay are primarily associated with changes in DHA and AA levels.

To further investigate the role of FA metabolism, human protein sequences involved in FA biosynthesis were retrieved from the UniProt database (Table 2).^1^ These proteins play essential roles in FA biosynthesis, metabolism, and regulation. Additionally, network analysis of DHA and AA was conducted using the FELLA package to explore the molecular interactions between KEGG pathways, enzymes, modules, and reactions. A metabolic regulation network was constructed, including 16 enzymes, 1 module, 25 reactions, and 10 KEGG metabolic pathways (Figure 5E). The top two related pathways identified were the PI3K-Akt signaling pathway and AA metabolism.

**Table 2:** human protein sequences involved in FA biosynthesis.

Entry	Entry name	Protein names	Gene names	Organism
P05413	FABPH_HUMAN	Fatty acid-binding protein, heart	FABP3	*Homo sapiens*
Q5TCH4	CP4AM_HUMAN	Cytochrome P450 4A22	CYP4A22	*Homo sapiens*
P09917	LOX5_HUMAN	Polyunsaturated fatty acid 5-lipoxygenase	ALOX5	*Homo sapiens*
Q03181	PPARD_HUMAN	Peroxisome proliferator-activated receptor delta	PPARD	*Homo sapiens*
Q8N9I5	FADS6_HUMAN	Fatty acid desaturase 6	FADS6	*Homo sapiens*
Q7L5A8	FA2H_HUMAN	Fatty acid 2-hydroxylase	FA2H	*Homo sapiens*

## 4 Discussion

SAMW is histopathologically characterized by myofiber atrophy and connective tissue proliferation, and ultrasound may reveal reduced muscle mass and a progressively blurred texture (20). GLCM metrics can quantify texture patterns, and prior evidence suggests their utility in predicting strength decline and identifying sarcopenia (21). In this study, bedside ultrasound was combined with texture analysis to delineate longitudinal changes in the structure and texture of the rectus femoris and dynamic metabolic/inflammatory profiles were integrated with untargeted metabolomics to explore metabolic phenotypes and potential mechanisms. Our findings indicate that the longitudinal decline trajectory of RF-CSA over the first week of ICU stay differentiated patients with SAMW from non-SAMW patients. Compared with single-time-point measurements, trajectory information more closely reflected the progressive muscle wasting that characterizes SAMW. Prior studies have also reported that acute skeletal muscle injury is common and rapidly progressive in sepsis and other acute proinflammatory states, sometimes evolving into persistent muscle damage (22–24); our longitudinal results reinforced this concept.

A decrease in CP and insulin with increasing GLU levels was observed, consistent with insulin resistance and impaired glycemic regulation. While GLU is essential for muscle function, persistently elevated plasma and tissue GLU levels can be cytotoxic, contribute to organ dysfunction, and promote skeletal muscle injury (25). Previous evidence has also shown markedly reduced insulin levels in patients with sarcopenia (26), and insulin resistance and muscle loss may lead to a vicious cycle (27). Concurrently, the rise in UCR and decline in TRF and CER highlighted a state that was both hypercatabolic and hypoanabolic. Furthermore, lipid alterations, characterized by decreased TC and HDL levels and increased LDL levels, suggested the occurrence of sepsis-associated lipid remodeling. Lipids are important substrates for muscle contraction, and FA and amino acids serve as signaling molecules that regulate metabolism and protein synthesis, thereby influencing muscle mass. Under surplus energy conditions, branched-chain amino acid accumulation can reduce insulin sensitivity and trigger insulin resistance and dyslipidemia (28). A recent meta-analysis has linked lipid metabolic risk factors to sarcopenia in older adults (29). Together, these observations suggest that SAMW may not merely be a transient complication of the inflammatory peak but rather a consequence of persistent metabolic imbalance. Longitudinal correlations supported this interpretation: RF-MLT was moderately negatively correlated with LDL, and RF-CSA was negatively correlated with TC, indicating that muscle phenotype changes parallel to lipid abnormalities. Moreover, the texture feature SDev correlated positively with GLU but negatively with IL-6, suggesting that texture features may capture tissue microenvironmental changes that are not necessarily tracked with inflammatory resolution. Thus, relying solely on declining inflammatory markers may underestimate ongoing muscle injury and underscore the need for concurrent metabolic and muscle phenotyping.

Metabolomics results suggested that, compared with controls, FA-related pathways were prominently altered at ICU admission and remained affected over the first week in septic patients with SAMW. Importantly, 29 metabolites displayed a progressive decline from the control to SAMW days 1 and 7, and the FA pathways were significantly enriched. DHA and AA were identified as key differential metabolites, and network analysis indicated their involvement in the PI3K-Akt signaling pathway and AA metabolism, suggesting that these metabolic alterations may be associated with the reprogramming of FA metabolism and regulatory changes in inflammatory networks in patients with sepsis accompanied by SAMW. FA play diverse roles in both pro- and anti-inflammatory processes. Hypoalbuminemia is frequently observed in patients with sepsis, which may disrupt the balance of FA-derived compounds, and has been associated with increased mortality (30). DHA (an *ω*-3 long-chain PUFA) has potential anti-inflammatory and pro-resolution effects (31), whereas AA (an ω-6 long-chain PUFA) has been reported to promote skeletal muscle cell growth via COX-2-dependent pathways and increase myotube protein content in C2C12 cells (32). Both DHA and AA have also been suggested to alleviate atrophy and inflammation via the inhibition of NF-κB nuclear translocation in experimental settings (33, 34). AA supplementation may restore protein synthesis under glucotoxic conditions and suppress hyperglycemia-induced inflammation and ROS production, thereby enhancing myogenesis (35). Consistent with this notion, low plasma DHA levels have been associated with reduced physical performance in older adults and DHA supplementation may improve their physical activity capacity (36, 37). Lipid metabolism may also have important potential for prognostication and precision therapy in sepsis (38).

Limitations included modest sample size and a limited SAMW subgroup, which may have affected the estimation of stability and generalizability. Different thresholds may lead to different estimates of the prevalence of SAMW. Larger multicenter studies are needed to develop standardized ultrasound-based diagnostic criteria for SAMW. In this study, untargeted metabolomic analysis was performed only in patients with SAMW and matched healthy controls. The absence of metabolomic data from the NSAMW group prevented us from determining whether these metabolic alterations were specific to SAMW or reflected broader sepsis-related metabolic dysregulation. Larger studies including both SAMW and non-SAMW patients with sepsis are needed to validate these findings and define SAMW-specific metabolic signatures. Some data were missing because patients were discharged from the ICU or died before follow-up assessments. Although linear mixed-effects models were used under the MAR assumption, missingness related to death may reflect a partially missing-not-at-random mechanism. Therefore, potential selection bias cannot be fully excluded. The key differential metabolites identified in this study (DHA and AA) require independent validation in future studies. Although nutritional therapy followed ESPEN guidance, detailed protein/calorie delivery, corticosteroid exposure, vasopressor burden, sedation duration, and mobilization intensity were not fully incorporated into the primary model. These exposures may influence muscle wasting trajectories and should be integrated into future studies.

## 5 Conclusion

By integrating longitudinal bedside ultrasound texture features with dynamic inflammatory and metabolic profiling, we propose an actionable clinical framework. Despite initial improvement in inflammatory markers during sepsis, clinicians should remain vigilant for rapid muscle loss indicated by RF-CSA and ongoing metabolic derangement reflected by lipid remodeling. In summary, although systemic inflammation gradually decreased during the first week of the ICU stay in patients with sepsis, those who developed SAMW showed persistent loss of muscle mass, which may be associated with metabolic dysregulation.

## Data Availability

The metabolomics data have been deposited in the MetaboLights repository under accession number MTBLS14997. The repository link is: https://www.ebi.ac.uk/metabolights/MTBLS14997. Further inquiries can be directed to the corresponding author.

## Footnotes

1
https://www.uniprot.org/

## Glossary

SAMW: Sepsis-associated muscle wasting
ICU: Intensive care unit
RF-CSA: Rectus femoris cross-sectional area
RF-MLT: Rectus femoris muscle layer thickness
GLCM: Gray-level co-occurrence matrix
ICUAW: ICU-acquired weakness
NSAMW: Non-sepsis-associated muscle wasting
PCA: Principal component analysis
PLS-DA: Partial least squares-discriminant analysis
VIP: Variable importance in projection
FC: Fold changes
DEMs: Differentially expressed metabolites
APACHE II score: Acute physiology and chronic health evaluation
SOFA score: Sequential organ failure assessment
NUTRIC: Nutrition Risk in Critically Ill
CP: C-peptide
GLU: Random glucose
TC: Total cholesterol
HDL: High-density lipoprotein
LDL: Low-density lipoprotein
RBP: Retinol-binding protein
TRF: Transferrin
CER: Ceruloplasmin
UCR: Urea-to-creatinine ratios
hs-CRP: C-reactive protein
IL-6: interleukin-6
FA: Fatty acid
TCA cycle: Citrate cycle
UFA: Unsaturated fatty acids
DHA: Docosahexaenoic acid
AA: Arachidonic acid
SDev: Standard deviation
ENT: Entropy
VAR: Variance
HOM: Homogeneity
CON: Contrast
UHPLC–MS/MS: Ultra-high-performance liquid chromatography–tandem mass spectrometry
